# Death of Dr. B. F. Wendel

**Published:** 1853-01

**Authors:** 


### DEATH OF DR- B. F. WENDEL.

We have just received the painful intelligence of the death of this promising young physician, which occured at Harbor Island, on the 18th of December. Dr. Wendel graduated in the University of Louisville, in 1847, and shortly afterwards went to New York, where he obtained a situation in Bellevue Hospital, Ward's Island, and continued to labor among the diseased emigrants until compelled to retire in the spring of 1848 by his own declining health. After a few months residence in St. Louis, his desire to improve still further his knowledge of practical medicine induced him to return to New York, where he became engaged in the Hospital on Blackwell's Island. From New York he went to the Bahama Islands in 1849, and fell a victim to the Yellow Fever while toiling night and day to rescue those around him from the destroyer. A friend at Harbor Island gives the following account of his last days in the midst of the fearful epidemic by which he was surrounded:

"It was the request of your poor friend, Dr. Wendel, that I should write to you and acquaint you of his death. We have had Cholera raging amongst us for the last nine weeks.—You who know these Islands can easily imagine the fatigue the medical officer of this place must have undergone. Dr. W. had one slight attack of Cholera, but he was so anxious to be again at his duties, that he remained only one day and a half at home. Within a very short time he had an attack of fever, again in spite of the entreaties of his friends he ventured out too soon, and at another short interval had another but slighter attack of fever. These repeated illnesses must have materially impaired his strength, but his unwearying benevolence, his sympathy for the sufferings of those around him, made him utterly forgetful of himself. He had no peace, no rest at home, for the people came in dozens for medicine and advice. One day he looked so thoroughly exhausted; he said 'I never passed such a dreadful time, and if I cannot get sleep I must die.' All night the sound of his horse's feet could be heard amid rain, wind, thunder and lightning."

In the midst of such labors he was seized with yellow fever and carried off in a few days. Dr. Wendel was a thoroughly educated and skilful physician, and possessed of every noble and manly virtue.

